# Clinical Evaluation of Short (6 mm) and Longer Implants Placed Side by Side in Posterior Partially Edentulous Area: A 3-Year Observational Study

**DOI:** 10.1155/2023/9086628

**Published:** 2023-07-07

**Authors:** Masahiro Shimogishi, Sawako Kawakami, Noriko Tachikawa

**Affiliations:** Department of Regenerative and Reconstructive Dental Medicine, Graduate School of Medical and Dental Sciences, Tokyo Medical and Dental University, Tokyo, Japan

## Abstract

**Background:**

Short implants have been proposed as an alternative solution for the rehabilitation of atrophic posterior region.

**Purpose:**

To compare the clinical outcomes between 6 mm short implants and conventional implants placed under similar conditions of bone quality and occlusal loading.

**Materials and Methods:**

Nine patients received atone 6 mm implant and one standard-length (8 mm length or longer) implants in a total of 10 partially edentulous areas. Implants were left submerged for 3–6 months healing period and the screw-retained splinted prostheses were delivered. When the provisional or final restoration was placed, and at each year after loading, standardized intraoral radiograph was taken for themarginal bone level (MBL) changes around the implants. Subsequently, the patients were recalled for the clinical examination evaluating the implant survival, sulcus bleeding index, suppuration, and the incidence of prosthetic complications at every 6 months after the definitive crown delivery. The observation period was continued to 3 years (mean follow-up was 3.4 ± 0.3 years) after functional loading.

**Results:**

Nine patients (10 short implants and 10 standard length implants) were selected in this study. Cumulative survival rates of the short implants and standard-length implants were 100% in both groups, and no biological and prosthetic complication were found in 3 years observation period. Cortical bone thickness of implant insertion sites was 1.39 ± 0.45 mm at short implants and 1.38 ± 0.69 mm at standard-length implants, and trabecular bone computed tomography values of implant insertion sites was 424.1 ± 290.1 at short implants and 410.9 ± 267.9 at standard-length implants. The MBL changes were −0.30 ± 0.71 mm at short implants and −0.19 ± 0.78 mm at standard-length implants at 3 years follow-up visit. No significant difference was found in the average of MBL changes between implant length.

**Conclusions:**

Within the limits of this study, it can be concluded that 6 mm short implants in a posterior edentulous region showed excellent results compared with conventional implants.

## 1. Introduction

Dental implant treatment became one of the predictable treatments for partially and totally edentulous patients. Several clinical investigations have been conducted in decades and reported that implant treatment achieved remarkable results in esthetics, speech, and mastication [1–3]. Development of implant design and surface characteristics achieved excellent performancein 10 years follow-up [4].

When dental implants are placed into jawbone, adequate bone quality and quantity are required around the implant insertion site. In the early days of implant dentistry, use of longer implants as much as possible was needed because of sufficient primary stability and bone-to-implant contact [5, 6]. However, insufficient bone height for standard-length implants can often be seen in the edentulous region of mandible and maxilla due to postextraction atrophy. Moreover, available locations for implant placement in the remnant bone are limited by anatomical limitations, such as mandibular canal, nasal cavity, and maxillary sinus. These circumstances make predictable dental implant treatment more complicated.

Therefore, bone augmentation materials and techniques have been invented and developed for widening dental implant applications. Numerous studies have confirmed the predictability of bone augmentation techniques for atrophic alveolar bone reconstruction [7–10]. However, these techniques still have some drawbacks in terms of high costs, prolonged treatment times, high- skill requirement, and the risk that recipient and donor site suffer from postoperative complication [11–16].

In contrast, some clinical studies reported that short implants (<8 mm length) showed comparable clinical outcomes to conventional length implants with bone graft [17–19]. The use of short implants may be an alternative solution for patients with insufficient bone volume by avoiding some disadvantages due to complicated bone grafting procedure and postoperative donor site morbidity. However, the risk for bone resorption in short implants is more critical than in longer implants, and actually it remains controversial issue in long-term clinical outcomes of short implants [20–23]. In a recent study, a tendency toward decreased survival rate was shown in short implants at 5-year observation [23], on the other hand, several other meta-analysis failed to demonstrate significant difference in the length of implant [20–22]. When it comes to marginal bone level (MBL) change during prosthetic loading, short implants showed less bone loss with statistical significance [23].

There are several concerns that may affect long-term clinical outcomes of dental implants: implant macro design and surface microstructure (length, diameter, surface topography, and chemistry), recipient site (bone quality, quantity, general condition, and medication), prosthetic restoration (single crown or splinted and cement or screw retention), and treatment procedure (bone graft and loading protocol). Several different designs of clinical studies were conducted to investigate reliability and effectiveness of short implants in consideration of the other factors which may contribute to their results. Several studies evaluated the performance of short and conventional implants in sites allowing the placement of both types of implants [24, 25]. Another research compared the use of short implants to conventional implants in combination with vertical bone augmentation procedures in split-mouth designs [17]. However, it is difficult to assess only the effect of implant length after excluding the other factors such as local bone quality, surgical procedures, and occlusal loading.

The aim of this study was to compare the clinical outcomes between 6 mm short implants and conventional implants placed under similar conditions of bone quality and occlusal loading.

## 2. Materials and Methods

This open label observational clinical study was carried out between May 2015 and December 2021 at the Dental Implant Clinic, Dental Hospital, Tokyo Medical and Dental University. The ethical approval for this study was received from the Ethical Committee of Tokyo Medical and Dental University (D2015-568). The manuscript was described in accordance with the STROBE guidelines.

### 2.1. Study Population

**Figure 1:**
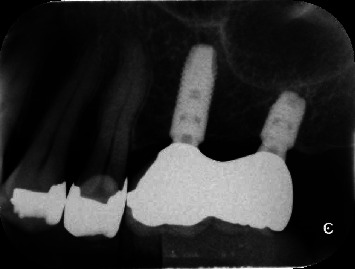
Intraoral radiographs showing after 3 years of loading. One short implant and one standard-length implant were placed next to each other.

Patients missing two consecutive teeth in their posterior maxilla or mandibles and having one tooth gap which needed to have a short implant (Figure 1 available bone height between 6 and 8 mm and ridge width ≥6 mm) were included. All patients were required according to the inclusion and exclusion criteria (Table 1) and signed the informed consent forms before registering for this study. Sample size calculation was performed based on previous bone loss data [26]. It was estimated that a total of 14 implants were required for two groups with *α* error of 0.05 and power of 80%. Preliminary screening including a clinical examination and radiological assessment of alveolar bone volume with computed tomography (CT) were performed.

### 2.2. Surgical Protocol

All patients received one short (4.0 mm width x6 mm length) implant and one standard-length (4.0 mm width x8 mm length or longer) implant (Astra Tech Implant System, Dentsply Sirona Implants, Sweden) next to each other (Figure 1). Implant placement was performed following the two-stage protocol, according to the manufacturer's guidelines. All implants had to reach a minimum stability of 15 N·cm. After surgery, patients were administered antibiotics (amoxicillin, 750 mg), analgesics at each patient's disposal for use (loxoprofen sodium, 60 mg or acetaminophen, 500 mg), and mouthwash (0.2% benzethonium chloride solution). Patients were instructed to refrain from tooth brushing at the surgical site. Suture removal was performed after 7–10 days, and patients were reinstructed keeping good intraoral hygiene.

### 2.3. Prosthetic Protocol

Implants were left submerged for 3–6 months healing period before surgical exposure. After the second stage surgery, an impression at the implant level was taken with vinyl-polysiloxane impression material (Imprint 2; 3 M Company, Minnesota, USA) using impression coping and customized impression trays. UniAbutments were connected to the implants, and screw-retained splinted polymethyl methacrylate based provisional restorations were delivered. About 3 to 6 months after provisional restorations delivery, final screw-retained splinted restorations were inserted.

### 2.4. Implant Success Rate and Radiographic Evaluation of MBL

**Figure 2:**
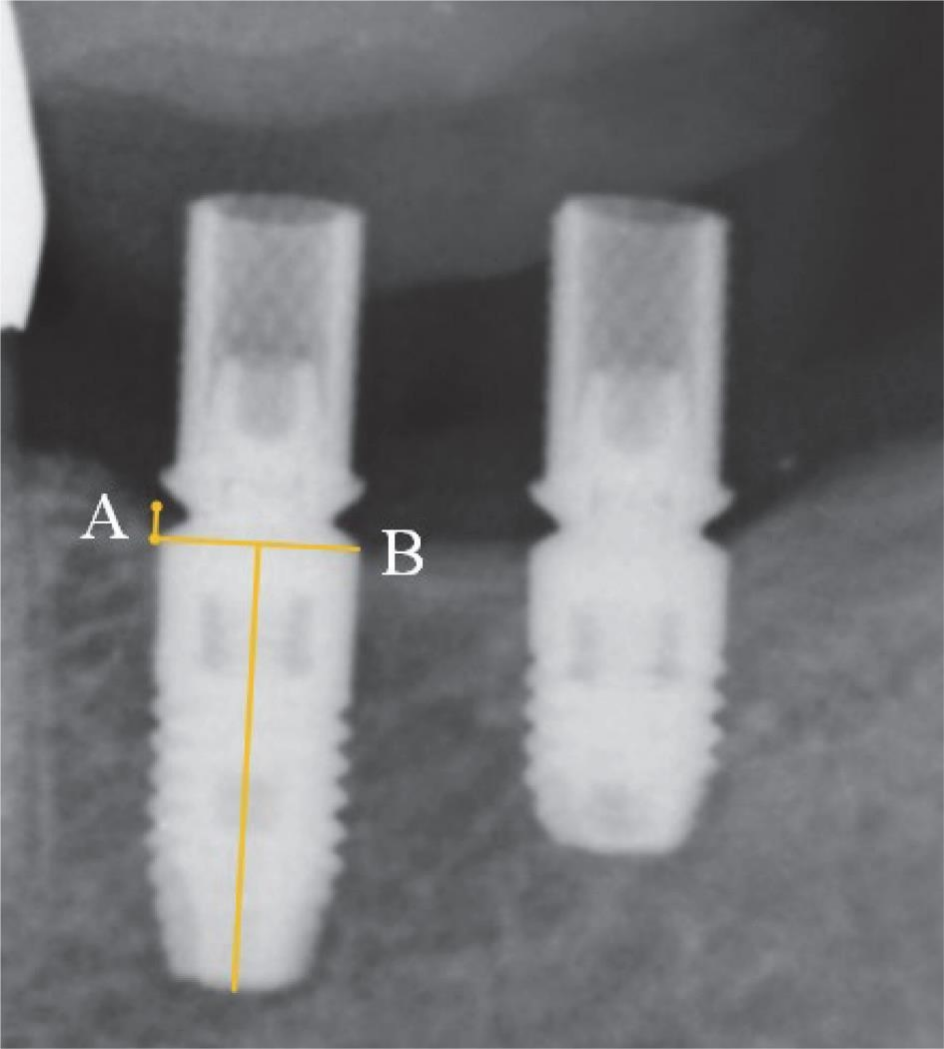
Method of measuring the radiographic marginal bone levels at the mesial (A), and distal (B) aspects of the implant platform.

**Figure 3:**
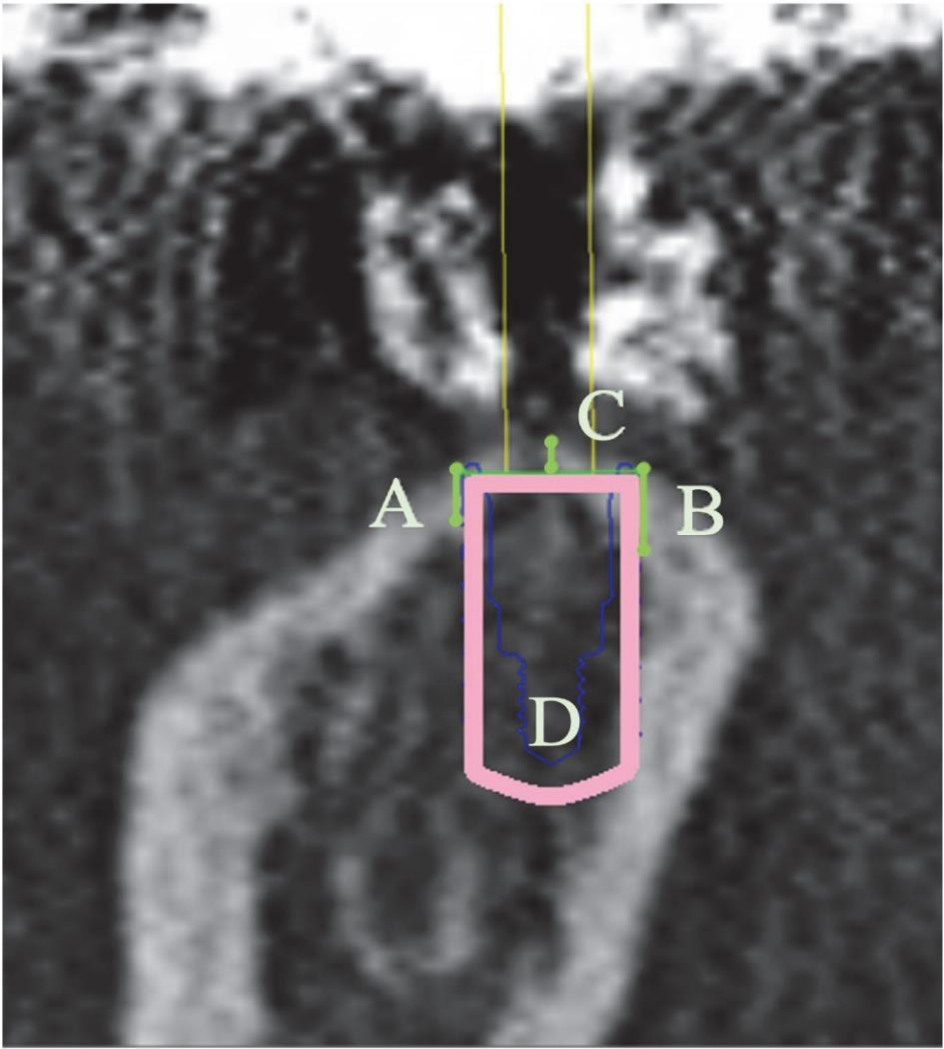
Thickness of the cortical bone at the site of implant placement on each cross-sectional computed tomography image. The cortical bone surface surrounding the implant model was measured at 3 points: the buccal (A), center (C), lingual, or palatal (B) sides, and the trabecular bone CT value of the implant placement site was measured (D).

Immediately after provisional restorations delivery, the baseline clinical examination was performed, and an intraoral radiograph was taken for the initial MBL around the implants. After final restoration delivery, the patients were scheduled for regular maintenance at every 6 months. The clinical examinationsof implant survival, occlusion, sulcus bleeding index [27], and the incidence of prosthetic complications were continued to 3 years after functional loading. Implant survival was defined as retention of implants regardless of complications [28]. Implant success was defined based on the previous reviews [29, 30]. As definition of a successful implant, there was neither an annual bone loss of more than 0.2 mm, a total bone loss of more than 0.6 mm during functional loading nor peri-implant mucosal inflammation. The success of implant restoration was defined as no incidence of prosthetic complications occurred. The diagnosis of peri-implant health condition was performed based onthe consensus report of the 2017 World Workshop on the Classification of Periodontal and Peri-Implant Diseases and Conditions [31]. When the provisional or final restoration was placed, and at each year after loading, standardized intraoral radiograph was taken with a film holder and cone alignment guide. According to the method of Chaytor [32], the marginal bone height was measured from the digital radiograph and determined as the distance from reference points on each side of implant platform to mesial and distal interproximal bone (Figure 2). The known implant length was used for calibration of the digital radiographs. MBL changes were determined as the difference in the marginal bone height at the average of mesial and distal aspects from provisional restoration delivery to 1–3-year postloading. Furthermore, cortical bone thickness and trabecular bone CT value of the implant placement sites were measured from each cross-sectional preoperative CT image [33]. The cortical bone thickness and trabecular bone CT value at the CT image of the virtually placed implant were calculated at the average of three points measurements (Figure 3 center of the implant, buccal, and lingual sides of the implant). The digital radiograph and CT image were evaluated for MBL changes and cortical bone thickness with image processing software (ImageJ 64; National Institute of Health).

### 2.5. Statistical Analysis

The normal distribution of peri-implant MBL changes, cortical bone thickness, and trabecular bone CT value in each group were confirmed by the Shapiro–Wilk test. The comparison between two groups of the mean MBL changes from the baseline to 1–3 years after loading was carried out using paired sample *t*-test. The correlation between cortical bone thickness, trabecular bone CT value, and MBL changes was measured by Pearson correlation coefficient. The significance level was set to *P* = 0.05, and all the statistical comparisons were carried out using IBM SPSS version 23 software for windows.

## 3. Results

### 3.1. Patient Population and Site Characteristics

Table 2 presents the details of patients in this study. The study population consisted of nine patients, two males, and seven females, with a mean age of 62.1 years (ranging between 53 and 72). They were treated from May 2015 to June 2018 and were followed-up to 3 years after prosthesis loading. Six patients received implant treatment in mandible and three in maxilla. A total of 20 implants, 10 short implants with a length of 6 mm, and 10 standard-length implants with a length of 8, 9, or 11 mm were inserted in the molar/premolar region. Nine short implants were placed in the molar region and one in the premolar region. Eight of the standard-length implants were placed in the molar region and two in the premolar region. No patients dropped out during the 3-years follow-up period.

### 3.2. Implant Success Rates and Complications

All the inserted implants were osseointegrated successfully, and no suppuration or mobility of the implants were observed during the 3 years follow-up period (100% survival rate). However, slight bleeding on probing (sulcus bleeding index score 1) occurred in two short implants and one standard-length implants of three patients at 3-year follow-up visit. These three patients received oral hygiene instructions and were reconfirmed that optimum level of oral hygiene was achieved and the symptoms of inflammation disappeared around the implants. Throughout the observation period, an annual bone loss of more than 0.2 mm occurred in three short implants and four standard-length implants, and a total bone loss of more than 0.6 mm during functional loading occurred in one standard-length implant. No other biological or prosthetic complications including fracture or loosening of retaining abutment/prosthetic screws, and chipping or fracture of the superstructure material were occurred during the observation period (Table 3). Therefore, tenset of short implants and standard-length implants were placed in nine patients, resulting in an overall postloading success rate of 70% and 60%, respectively.

### 3.3. Peri-Implant Marginal Bone Level Changes

The MBL changes in relation to the implant length 1–3 years after loading are shown in Table 4. Average change of the MBL around short implants and standard-length implants after 3 years were −0.30 ± 0.71 and −0.19 ± 0.78 mm, respectively. Though there was no significant difference in the average of MBL changes between implant length (*P* = 0.43), this finding indicates slight bone gain was occurred around both short and standard-length implants.

The results of the cortical bone thickness and trabecular bone CT value at implant placement sites are shown in Table 5. Forbone characteristics, no significant difference was found in cortical bone thickness (*P* = 0.98) and trabecular bone CT value (*P* = 0.74) between the short implant and standard-length implant group. Pearson correlation showed no significant correlation between the cortical bone thickness, trabecular bone CT value and the MBL changes 3 years after loading (*P* = 0.68 and 0.26, respectively).

## 4. Discussion

In the present study, we compared the clinical outcomes between 6 mm short implants and conventional length implants placed under similar conditions of bone quality and occlusal loading. The results obtained in this study show that short implants with a TiO_2_ blasted, fluoride modified surface in the maxillary or mandibular posterior region have performance comparable to conventional implants for 3 years functional loading. No early nor late failure was occurred during observation period, and there was no statistical difference in MBL changes between short implants group and standard-length implant group.

At 3 years after functional loading, the mean survival rates were 100% in both groups, and the mean MBL changes amounted to −0.30 ± 0.71 mm in short implant group and −0.19 ± 0.78 mm in standard-length implant group. The results of the present study are comparable with clinical outcomes of TiO_2_ blasted, fluoride modified implants showed acumulative survival rate of 100% and mean MBL change of −0.16 mm during the loading period of 5 years [34]. On the other hand, in the present study, ten set of short implants and standard-length implants were placed in nine patients, resulting in an overall postloading success rate of 70% and 60%, respectively. Although there was no significant difference between the groups, it resulted in a relatively low-success rate in the present study. The difficulty in cleaning the prosthesis may affect the prevalence of peri-implant disease and be associated with the acceleration of the marginal bone loss [35], and nonsplinted restorations were preferred by patients due to ease of hygiene [36]. With regard to prosthetic success rate, no prosthetic complications occurred throughout the 3-years functional loading, resulting in a success rate of implant restoration of 100%. A recent study suggested that splinted restorations were associated with a decreased rate of implant failure [37]. Splinted prosthesis may increase stability of super structure and reduce the stress transferred to peri-implant marginal bone.

In the present study, no significant correlation was found in the cortical bone thickness, trabecular bone CT value and the MBL changes. In addition, no difference found in bone quality at implant placement sites between short and conventional length group. Especially in the case of poor bone quality, dental implant clinicians prefer to use long length and wide diameter implants as much as possible because primary implant stability was influenced not only implant size and geometry [6, 38] but also by cortical bone thickness and trabecular bone density [33, 39]. Meanwhile, it has been controversial whether bone quality around implants influences MBLchanges under functional loading, especially in cervical regions. Dias et al. [40] reported no significant correlation between cortical bone thickness and changes on marginal bone height over time, but on the other hand Ibañez et al. [41] revealed that poor bone quality was associated with less loss of marginal bone around implants. Considering stress distributions around implants in function, three-dimensional finite element (3D-FE) analyses reported the highest stress concentrations were found in the cortical bone area around the neck of the implant [42–45]. Moriwaki et al. [46] investigated correlation between peri-implant cortical bone stress distribution and implant length, bicortical anchorage and sinus augmentation by using 3D-FE model of a maxillary posteriorand concluded bicortical anchorage may work as a stress-reducing factor, which can contribute to reducing peri-implant bone resorption. Even though the results of the present study cannot be compared with those studies due to the differences in the study designs (two implants were splinted by the fixed prostheses), it may be suggested that 6 mm-long implants may be considered an adequate length at least for 3 years functional loading.

In the present study, we placed short implant and conventional implant in positions adjacent to each other, under similar conditions of cortical bone thickness, and delivered the splinted prostheses to equalize occlusal force distribution. To date, published studies have evaluated the clinical outcomes of dental implants with split-mouth designs to remove a lot of intersubject variability from the estimated treatment effect. However, split-mouth designs have several limitations such as carry-across effects, period effects, and difficulty in recruiting patients fulfill inclusion criteria [47], and moreover, in the case of the present study, there were uncontrollable factors such as laterality of local bone quality and varying occlusal stress according to the tooth position [48]. When it comes to conventional length implants, no significant difference was found in MBL changes between splinted and nonsplinted restorations, especially in the posterior region [37, 49], and a study showed that the interposition of an occlusal splint reduces the stress generated in the long axis of implant [50]. Moreover, a FEA analysis revealed a decrease and equalize in strain values on the implants using occlusal splint in the premolar and molar region [51]. Thus, the splinted implant-supported fixed prostheses were delivered to equalize occlusal stress distribution in this study. However, such favorable effects should lead to extend longevity of each splinted implants beyond expectation. As a matter of fact, splinted protheses were not affected a marginal bone change and prosthetic complication rates, but associated with a decreased rate of implant failure [37]. Therefore, it is still unclear whether single short implants will show equivalent outcomes compared with conventional implants, even though previous studies demonstrated the high-survival rates and minimum marginal bone loss of single short implants [52, 53]. In addition, this study has limitation included its small sample size, which might be the reason for the lack of significant differences between the groups. Therefore, the generalizability of our results is limited. Further well-designed studies with a larger number of patients are required to analyze biomechanical prognosis of short implants.

## 5. Conclusions

Clinical outcomes of 6 mm short implants and conventional implants placed under similar conditions of bone quality and occlusal loading were analyzed in terms of MBL changes, survival rates, and the other biological or prosthetic complications. Within the limits of this study, it can be concluded that short implants in a posterior edentulous region showed excellent results compared with conventional implants.

## Figures and Tables

**Table 1:** Selection criteria.

Inclusion criteria	Exclusion criteria
Missing premolars and/or molars Good general health Provide written informed consent	Oral infections or uncontrolled periodontal disease Parafunctional occlusal habits No opposing teeth Uncontrolled diabetes mellitus Immunodeficiency History of irradiation of head and neck Psychological or psychiatric disorders Pregnancy or lactation Requirement for bone augmentation

**Table 2:** Patient characteristics.

Patient ID.	Age	Sex	Implant site (short)			Implant site (conventional)			Observation period (years)
			Implant site	Bone height (mm)	Implant size (mm)	Implant site	Bone height (mm)	Implant size (mm)	
1	56	F	27	6.5	4 × 6	26	11.2	4 × 11	3.2
2	65	F	37	6.2	4 × 6	36	10.7	4 × 9	3.9
3	53	F	26	6.2	4 × 6	25	8.2	4 × 8	3.3
4	72	M	16	6.3	4 × 6	15	10.7	4 × 9	3.3
5	71	F	37	6.6	4 × 6	36	11.2	4 × 11	3.3
6	59	F	47	6.6	4 × 6	46	9.5	4 × 9	3.3
7	56	M	37	6.6	4 × 6	36	9.5	4 × 9	3.6
8	59	F	45	7.1	4 × 6	46	8.3	4 × 8	3.3
9	68	F	37	7.8	4 × 6	36	8.3	4 × 8	3.7
			47	7.8	4 × 6	46	8.3	4 × 8	3.0

**Table 3:** Data related to implant success, biological, and prosthetic complications.

		Short implant		Conventional implant	
		Maxilla	Mandible	Maxilla	Mandible
Annual bone loss ≥ 0.2 mm	Mesial	0	3	0	3
	Distal	0	2	1	1

Total bone loss ≥ 0.6 mm	Mesial	0	0	0	1
	Distal	0	0	0	0

Sulcus bleeding index ≥1		0	2	0	1

Suppuration		0	0	0	0

Prosthetic complications		0	0	0	0

**Table 4:** Marginal bone level changes from baseline to 1 year and 3 years of loading.

			Short implant		Conventional implant		*P*
			Mean	SD	Mean	SD	
1 year MBL changes (mm)	Maxilla	Mesial	−0.81	1.40	−0.87	1.14	0.94
		Distal	−1.07	1.15	−0.37	0.86	
		Average	−0.93	1.27	−0.62	1.00	
	Mandible	Mesial	0.58	0.47	0.11	0.58	0.64
		Distal	−0.31	0.90	−0.07	0.59	
		Average	−0.13	0.63	0.02	0.54	
	Total	Mesial	−0.20	0.87	−0.18	0.86	0.31
		Distal	−0.54	0.98	−0.16	0.64	
		Average	−0.38	0.88	−0.17	0.72

3 years MBL changes (mm)	Maxilla	Mesial	−0.82	1.37	−1.09	1.01	0.74
		Distal	−1.08	0.74	−0.72	1.00	
		Average	−0.95	1.05	−0.91	1.00	
	Mandible	Mesial	−0.03	0.21	0.12	0.46	0.50
		Distal	−0.02	0.42	0.11	0.50	
		Average	−0.03	0.28	0.11	0.46	
	Total	Mesial	−0.27	0.77	−0.25	0.84	0.43
		Distal	−0.34	0.71	−0.14	0.74	
		Average	−0.30	0.71	−0.19	0.78	

**Table 5:** Cortical bone thickness and trabecular bone CT value of the implant placement sites.

			Short implant		Conventional implant		*P*
			Mean	SD	Mean	SD	
Cortical bone thickness (mm)	Maxilla	Buccal	0.83	0.06	0.97	0.31	0.40
		Central	0.73	0.06	1.33	0.85	
		Lingual	0.80	0.40	0.83	1.53	
		Average	0.79	1.68	1.04	0.41	
	Mandible	Buccal	2.21	1.72	1.61	0.32	0.36
		Central	2.26	1.98	1.60	0.54	
		Lingual	1.87	1.76	1.40	0.62	
		Average	2.11	1.80	1.54	0.41	
	Total	Buccal	1.42	0.43	1.50	0.76	0.98
		Central	1.52	0.61	1.40	0.71	
		Lingual	1.23	0.58	1.25	0.76	
		Average	1.39	0.45	1.38	0.69	

Trabecular bone CT value (HU)	Maxilla		307.2	143.3	338.2	262.0	0.72
	Mandible		474.2	331.2	442.1	284.6	0.48
	Total		424.1	290.1	410.9	267.9	0.73

## Data Availability

Access to data is restricted for ethical and privacy considerations.

## Additional Points

*Summary Points*. *What is known*: Several systematic reviews suggested that short implants and conventional implants have the equivalent survival rate and marginal bone maintenance. These reviews were based on the parallel-group or split-mouth design studies which compared short implants and long implants with bone graft. *What this study adds*: This study suggests that 6 mm length short implants and longer implants have the same survival rate and marginal bone maintenance in the situation of similar bone quality and occlusal loading.
